# CAR T cell therapy for T cell leukemia and lymphoma: latest updates from 2022 ASH Annual Meeting

**DOI:** 10.1186/s13045-023-01406-8

**Published:** 2023-03-05

**Authors:** Qiusha Huang, Xiao-hui Zhang, Delong Liu

**Affiliations:** 1 Peking University People’s Hospital, Peking University Institute of Hematology, Beijing Key Laboratory of Hematopoietic Stem Cell Transplantation, National Clinical Research Center for Hematologic Disease, Beijing, China; 2 New York Medical College and Westchester Medical Center, Valhalla, NY 10595 USA

**Keywords:** CAR T, CD3, CD7, T cell leukemia, Lymphoma

## Abstract

Due to the concern of fratricide, clinical development of CAR T cells for the therapy of T cell malignancies lags behind that for B cell malignancies. Attempts are being made to revise T cell biomarkers so that the re-engineered CAR T cells can target T cell malignancies. CD3 and CD7 are the two pan-T cell surface biomarkers that have been either knocked out or knocked down through genome base- editing technology or by protein expression blockers so that the re-engineered T cells can target T cells without fratricide. We summarized several latest reports on the CAR T cells for the therapy of T cell leukemia /lymphoma from the 2022 ASH Annual Meeting, with latest updates on clinical trials of TvT CAR7, RD-13-01, and CD7 CART.

To the editor

Attempts are being made to revise T cell biomarkers so that the re-engineered CAR T cells can target T cell malignancies without fratricide [1]. CD3 and CD7 are the two pan-T cell surface biomarkers that have been either knocked out or knocked down through genome base- editing technology or by protein expression blockers so that the re-engineered T cells can target T cells without fratricide [2, 3]. We summarized several latest reports on the CAR T cells for the therapy of T cell leukemia /lymphoma (TCLL) from the 2022 ASH Annual Meeting (ASH2022).

## CD7 targeted CAR T cells by genome editing

The abstract 2001 reported a targeted base- editing (BE) technology that can mediate precise C → U → T conversion using CRISPR guided cytidine deamination [4] (Table 1). The BE-edited allogeneic CAR T cells have disruptions of TCR, CD7 and CD52 (BE-CAR7). Early data from the clinical trial, TvT CAR7 study, were reported at ASH2022 [4]. This phase I study was planned for children with relapsed /refractory (R/R) CD7 + T-ALL. The BE-CAR7 cells were used to induce negative measurable residual disease (MRD) prior to allogeneic hematopoietic stem cell transplant (alloHSCT) at day + 28 (Table 2). The first highly refractory T ALL subject treated had grade 2 cytokine release syndrome (CRS), grade I immune effector cell-associated neurotoxicity syndrome (ICANS). The patient (pt) successfully achieved MRD negativity, had no graft-vs-host disease (GVHD), and received alloHSCT.

**Table 1:** Properties of CAR T cells targeting T cell malignancies

Product	Genome editing	PEBL	Target	T cells	References
BE-CAR7	+	–	CD7	Off-the-shelf	[4]
RD-13-01	+	–	CD7	Off-the-shelf	[5]
PCART7	–	+ CD7	CD7	Donor	[6]
CD3PEBL	–	+ CD3	CD3	Off-the-shelf	[8]
CD3/7PEBL	–	+ CD3/7	CD7	Off-the-shelf	[7]

*PEBL* protein expression blocker

**Table 2:** Outcomes of clinical trials of CAR T cells targeting T cell malignancies

Product	BE-CAR7	RD-13–01	PCART7
Patients	1	10	20
Conditioning regimen	Fludarabine (150 mg/ m^2^), cyclophosphamide (120 mg/kg) and alemtuzumab (1 mg/kg)	Fludarabine (25–30 mg/m^2^/d), cyclophosphamide (300 mg/m^2^/d) and etoposide (100 mg/m^2^/d) on Day -6 to Day -3	
ORR/CR	1/1 MRD negative on day 28	8/10 (80%) CR on day 28	18/20 (90%) ORR at 3 months
PFS		4/6(66.7%) 315-day PFS	62.3% 1-year PFS
OS			60% 1-year OS
GVHD	0/1		8/20 (40%) grade 1–2 GvHD
CRS/ICANS	1/1 grade 2 CRS 1/1 grade 1 ICANS	9/10 (90%) grade 1 CRS, 1/10 (10%) grade 3CRS	2/20 (10%) grade 3 or higher CRS
Clinical trial number	ISRCTN15323014	NCT04620655	NCT04689659
References	[4]	[5]	[6]

*CAR* chimeric antigen receptor, *CRS* cytokine release syndrome, *CR* complete response, *GVHD* graft vs host disease, *ICANS* Immune effector cell-associated neurotoxicity syndrome, *MRD* measurable residual disease, *ORR* overall response rate, *PFS* progression free survival, *OS* overall survival

Another study reported data on a different CD7- genome edited alloCAR T cell product, RD13-01, in 10 patients with R/R TCLL [5]. On day 28, 8 pts reached complete remission (CR), and 7 of them had negative MRD. 6 patients in CR (including 1 MRD + CR) proceeded to alloHSCT. Among the 10 subjects, 4 had durable CR, 4 died, 2 had no response. Nine of the 10 pts had grade I and 1 with grade 3 CRS, 1 had ICANS. GVHD was not reported.

## CD7 targeted CAR T cells by protein expression blocking

By employing an IntraBlock technology that can retain CD7 intracellularly, thereby leading to downregulation of surface CD7 expression, CD7-targeted CAR T cells from either stem cell donors or new donors were engineered and infused to patients with R/R TCLL [3]. In a recent report of phase 2 data with a 11 -month median follow-up [6], the objective response rate (ORR) remained at 90% 3 months post-infusion for the 20 enrolled pts. The rate for one-year progressive-free survival was 62.3%, and overall survival rate at 1 year was 60.0% (95% CI, 38.5–81.5). There was 10% grade 3 or higher CRS. Grade 1–2 GVHD was at 40%.

CD7 protein expression blocker (PEBL) binds and anchors CD7 in the ER and Golgi apparatus, resulting in CD7 degradation, thus leading to downregulation of surface CD7 expression. One group constructed a dual CD3/CD7 PEBL to block both CD3 and CD7 expression, together with anti-CD7 CAR, the CAR T cells, PCART7, specifically targeted CD7 + T cells in vitro and in vivo [7]. Due to lack of TCR activation secondary to CD3 downregulation, GVHD was not observed. This approach avoids genome editing, though clinical trials are needed to verify the applicability in human.

## CD3 targeted CAR T cells by protein expression blocking

By using the CD3 PEBL mentioned above, anti-CD3 PEBL-CAR T cells were generated that have been shown to have potent and specific cytotoxicity against CD3 + target cells in both in vitro and in vivo models [8]. Again, GVHD was much reduced in the mouse model treated with the anti-CD3 PEBL-CAR T cells. These features make this novel anti-CD3 CAR-T cells suitable for the treatment of TCLL.

In summary, by genome knockout or by blocking protein expression of surface CD3 and /or CD7, it is feasible to avoid fratricide and target T cell malignancies through CAR T cells.

## Data Availability

The material supporting the conclusion of this study has been included within the article.

## Abbreviations

ASH: American Society of Hematology
ALL: Acute lymphoblastic leukemia
CAR: Chimeric antigen receptor
CRS: Cytokine release syndrome
ICANS: Immune effector cell-associated neurotoxicity syndrome
CR: Complete response
GvHD: Graft vs host disease
ORR: Overall response rate
PFS: Progression free survival
OS: Overall survival
